# Supplementary material for: Mixed-methods feasibility study of blood pressure self-screening for hypertension detection
Source: BMJ Open. 2019 May 29;9(5):e027986. doi: 10.1136/bmjopen-2018-027986 (PMC6549634; doi:10.1136/bmjopen-2018-027986)
Supplement: Supplementary data [file bmjopen-2018-027986supp001.pdf]

Supplementary data

Table 1 Unit costs used in the economic analyses

	Cost (£)	Cost per 5 minutes (£)	Source
	**Annual cost**		
Kiosk	4000	n/a	Study data
	**Cost per hour**		
GP	220	18.3	Curtis (2017)
Practice nurse	42	3.5	Curtis (2017)
HCNA (band 2)	23	1.9	Curtis (2017)

Table 2 Ethnicity of kiosk users.

Direct comparison between practices is difficult due to differences in coding methodology

	Practice A (N=60)	Practice B (N=126)
N ethnicity (%)	British or mixed British 14 (23.3)<br>Other White background 2(3.3)<br>Unknown 44 (73.3)	White 61 (48.4)<br>Asian 14 (11.4)<br>Black 11 (8.7)<br>Mixed 15 (11.9)<br>Unknown 24 (19.0)<br>Other 1 (0.8)

Table 3 Number of patients coded as receiving different methods of hypertension diagnosis at Practice A across both the control and intervention periods

For reference, the total number of patients diagnosed with hypertension at this site across these periods was 91. It was not possible to do a similar analysis at Practice B as all patients with an ambulatory BP code also had a home BP code, and vice versa.

	**Practice A (N=5138)**		
	Home BP	Ambulatory	Both
N patients coded as receiving method (%)	1 (0.02)	38 (0.7)	0 (0.0)
